# Significant risk of developing asbestos-related diseases in Japan's industries: An analysis of workers' compensation

**DOI:** 10.3934/publichealth.2025053

**Published:** 2025-11-04

**Authors:** Leli Hesti Indriyati, Masamitsu Eitoku, Naw Awn J-P, Taro Tamura, Narufumi Suganuma

**Affiliations:** 1 Department of Environmental Medicine, Kochi Medical School, Kochi University, Kochi, Japan; 2 Department of Occupational Health, Faculty of Medicine, University of Muhammadiyah Prof. Dr. Hamka, Jakarta, Indonesia; 3 Izumino Hospital Medical Corporation Bouchikai, Kochi, Japan

**Keywords:** asbestos, asbestos-related disease (ARD), mesothelioma, Japan, workers

## Abstract

**Background:**

Asbestos has been used in various industries, and prolonged exposure can increase the risk of asbestos-related disease (ARD). Although the use of asbestos has been prohibited in Japan since 2012, it was imported well into the 2000s. This study aimed to identify industries in Japan whose workers are at heightened risk of developing ARD.

**Methods:**

This study was based on a comprehensive analysis of fiscal year data from the Survey of Claims and Decisions on Benefits for Asbestos-Related Health Damage and calendar year data from the Status of Pneumoconiosis Health Management Implementation in Japan. Occupations associated with asbestos exposure risk were identified using the Japan Standard Industrial Classification (Rev. 14, 2023). The incidence rate ratio (IRR) was estimated using Poisson regression analysis.

**Results:**

This study includes 8,971,500 person-years from 2006 to 2022. Construction workers showed the strongest positive associations with all ARDs. Within the manufacturing sector, shipyard workers were particularly vulnerable to ARDs, although a notable exception to this was the positive correlation with asbestosis among workers in the ceramic industry (IRR 1.56; 95% *CI* 1.12–2.17). Mesothelioma is the most prevalent ARD over the past 17 years.

**Conclusions:**

This study suggests that, despite asbestos being banned in Japan for the past 13 years, cases of ARDs continue to occur in various industries, with construction and shipyard workers being at highest risk. Consequently, individuals with prior asbestos exposure require continuous monitoring to avoid potential adverse health consequences.

## 1. Introduction

Asbestos has been used in various sectors [1]–[5] because of its high tensile strength, non-flammable properties, thermal and electrical resistance, stability, and chemical resistance [1],[5]. In Japan, products containing asbestos are generally either building products, industrial asbestos products, or friction materials [5]–[7]. Building products account for the largest volume of asbestos [5],[7].

Most of the asbestos used in Japan was imported [6],[8]. Following World War II, Japan imported significant amounts of asbestos, reaching a peak of around 350,000 tons in 1974 [6],[8],[9]. Administrative recommendations and the voluntary actions of industry were not enough to halt the use of asbestos. In fact, in the late 1980s, asbestos imports into Japan increased again [5].

Asbestos imports into Japan started declining in 1989; however, Japan continued to be the world's top importer of asbestos until 1999 [10]. In 2004 and 2005, there was a noticeable decline and no asbestos has been imported since 2006 [5],[6],[8],[10]. The Japanese government prohibited the use of asbestos from 2006, with a total ban implemented in 2012 [5],[7],[8].

Japan ranks third nationally and regionally in fatalities resulting from occupational asbestos exposure, behind the United States and China [11]. In 1995, there were 500 documented fatalities from mesothelioma, a figure that consistently rose to 1504 by 2015 [8]. Nevertheless, prior to the ‘Kubota Shock' in 2005, when the Kubota company announced that a number of its employees had died as a result of mesothelioma and when other companies made similar announcements in subsequent days [7],[8],[12],[13], the Japanese government had progressively regulated the use of asbestos [5],[8],[14]. For example, the 1975 Ordinance on Prevention of Hazards Due to Specified Chemical Substances had strict measures to prevent occupational cancers due to asbestos exposure [5],[8],[14]. Asbestos-related diseases (ARDs), including non-malignant diseases and some cancers, were previously designated as eligible for workers' compensation in Japan under the Labor Standards Act [15].

Despite major reductions in the number of cases of ARDs in many countries, it is expected that ARDs will persist over the next decade [1],[16],[17]. In Canada and Australia, asbestos exposure has been identified in the majority of compensation claims for mesothelioma and lung cancer [2],[3]. In Japan, people who were exposed to asbestos in around 1970s and later developed ARD, such as mesothelioma, are now emerging [7]. By the end of March 2000, there were 197 compensation payouts for lung cancer [6]. Over the 18-year period from 1987 to 2004, there were approximately 500 compensation payouts for mesothelioma, with this number increasing by 500 in 2005 and by 1000 in 2006 due to the Kubota shock [8]. Furthermore, according to a 2022 report from the Ministry of Health and Welfare of Japan, there were 1079 newly approved compensation payouts for ARD to workers in Japan, a 6.6% increase over the previous year [18]. Thus, it is essential to identify workforce sectors with an elevated risk of ARD, to monitor disease prevalence over time and to identify still-actual occupational exposures to asbestos for preventive measures.

Currently, no study has quantified the incidence rate ratio (IRR) for ARDs in different workforce sectors in Japan. Thus, in the present study, we compared IRRs for ARDs in different industries to identify specific industries in which workers are at a heightened risk of developing ARD. This information could be used to inform future occupational epidemiological surveys and targeted preventive measures in these sectors.

## 2. Methods

### 2.1. Study setting

This study was based on a comprehensive analysis from fiscal year data of the Survey of Claims and Decisions on Benefits for Health Damage Caused by Asbestos and calendar year data from the Status of Pneumoconiosis Health Management Implementation in Japan. We collected the data from 2006 to 2022 as our observation period in years which refers to the timeframe of the newly reported cases. These reports are produced by the Japanese Government, which gets the data from the many industries that conduct specific health check-ups for their workers.

### 2.2. Classification of industries

Based on the literature [2],[6],[19],[20], the occupations at risk of asbestos exposure were identified from ARD compensation documents according to the Japan Standard Industrial Classification (JSIC; Rev. 14, 2023) [21]. The JSIC is divided into four categories: divisions, major groups, groups, and details (industries). There are 20 divisions, 99 major groups, 530 groups, and 1460 industries. The list of sectors was translated from Japanese into English. Each group of different sectors (based on JSIC) in the manufacturing industry was recognized at risk of asbestos exposure (Supplementary Table S1).

### 2.3. Study population

Because the number of workers specifically surveyed for asbestos exposure in Japan is unknown, we used the Status of Pneumoconiosis Health Management Implementation [22], which indicates the population of dust-exposed workers, as the population at risk. That report contains statistics on pneumoconiosis health examinations conducted regularly on dust-exposed workers as defined by the Pneumoconiosis Act. It is a comprehensive collection of information on major dust-related work in Japan, and we presumed that this population also includes workers exposed to asbestos. The total number of dust-exposed workers in each of the identified occupations and industries from 2006 to 2022 was used for analysis.

### 2.4. Cases of ARD

Japan has two categories of ARD compensation programs: workers' compensation and the Victim Relief Act, which compensates individuals with ARD who are not covered by workers' compensation [7],[8]. This study focused exclusively on workers' compensation, which pertains specifically to direct asbestos exposure.

For each ARD (lung cancer, mesothelioma, benign asbestos pleural effusion, and diffuse pleural thickening), the number of newly compensated cases was referenced from the annual records of ARD that had been approved for compensation through the workers' compensation system. The diagnosis criteria for all diseases of ARD are explicitly established, as they have already been defined by Ministry of Health, Labor, and Welfare [23].

Each person who received workers' compensation was categorized according to group and detail (industry) as per the JSIC. All compensation data for ARD in Japan from 2006 to 2022 were collected.

### 2.5. Statistical analysis

The IRR for ARD was calculated using a person-year approach. Person-time denominators for the calculations of incidence density were derived from the number of dust-exposed workers in the Status of Pneumoconiosis Health Management Implementation. The incidence rate (IR) is reported per 10,000 workers (population) in each industry, and is derived from the number of event cases for each disease divided by the population size. The IRR was stratified by the type of industry and calendar period, with exact 95% confidence intervals (*CI*s) being calculated from the Poisson distribution. In order to estimate IRRs, Poisson regression analysis was used. The population number was used as offset in our models. All analyses were performed using Stata statistical software.

## 3. Results

This study includes 8,971,500 person-years from 2006 until 2022. The IRRs for ARD by industry grouping are presented in Table 1. Construction workers had the strongest positive associations for all ARDs. Of the ARDs, greater estimates were observed for asbestosis (IRR 8.36; 95% *CI* 6.32–11.07), followed by lung cancer (IRR 8.05; 95% *CI* 7.34–8.84), among construction workers. Table 1 indicates that the manufacturing industry overall has weak associations with these ARDs. However, as indicated in Table 2, within the manufacturing industry, shipyard workers have the highest risk for almost all the ARDs. An exception to this was the positive association with asbestosis among workers employed in the ceramic industry (IRR 1.56; 95% *CI* 1.12–2.17).

**Figure 1.**
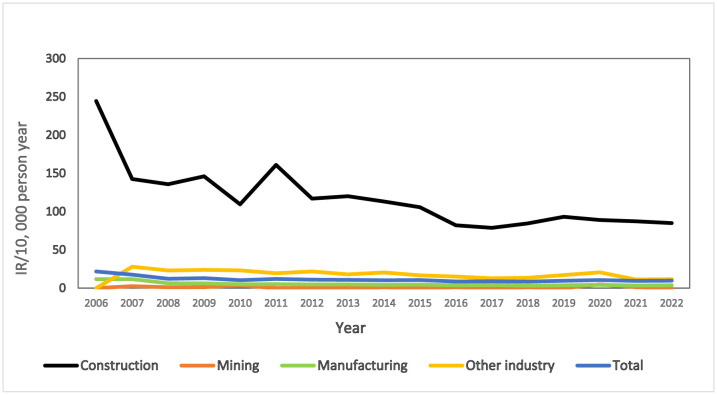
Incidence rate (IR) for mesothelioma according to industry from 2006 to 2022.

**Figure 2.**
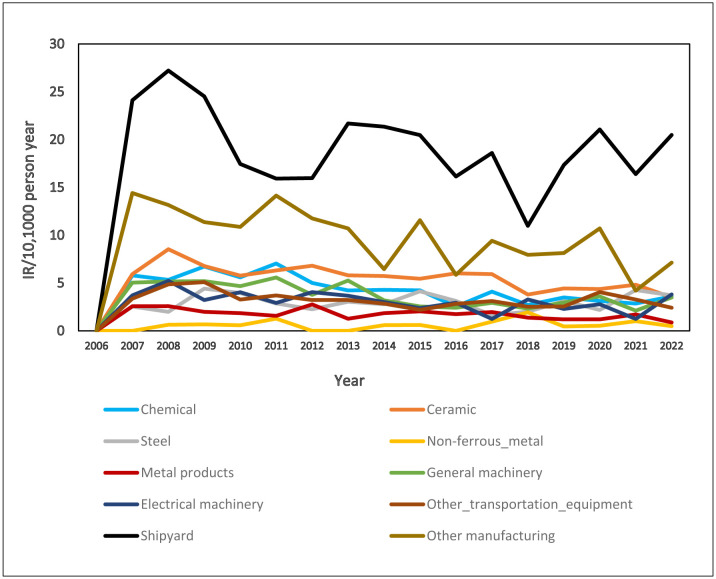
IR for mesothelioma in different sectors of the manufacturing industry.

Of all the ARDs recorded in the past 17 years, mesotheliomas accounted for the majority of all cases in all industries. Figure 1 shows the annual apparent IR for mesothelioma in all industry sectors. For all 17 years, construction workers had the highest IR for mesothelioma (Figure 1), with a peak in 2006 (IR 250) followed by a relatively stable IR for the rest of the period (IR 80–250). Within the manufacturing industry, shipyard workers had the highest IR, followed by workers in other manufacturing (as defined in Supplementary Table S1) and the ceramic industry (Figure 2).

**Table 1.** Risk of asbestos-related diseases according to industry.

	Observation period (years)*	Person-years	No. events	IRR (95% *CI*)
Lung cancer due to asbestos				
Construction	17	474,508	3940	8.05 (7.34–8.84)
Mining	16	135,711	7	0.05 (0.02–0.10)
Manufacturing	17	7,872,187	2695	0.33 (0.30–0.36)
Other industry	16	489,094	504	1.00
Mesothelioma				
Construction	17	474,508	5133	6.23 (5.79–6.70)
Mining	16	135,711	15	0.06 (0.04–0.11)
Manufacturing	17	7,872,187	3733	0.27 (0.25–0.29)
Other industry	16	489,094	849	1.00
Asbestosis				
Construction	12	380,855	433	8.36 (6.32–11.07)
Mining	12	102,280	1	0.07 (0.01–0.52)
Manufacturing	12	5,920,556	283	0.35 (0.26–0.47)
Other industry	12	404,556	55	1.00
Benign asbestos pleural effusion				
Construction	15	438,118	207	5.28 (3.79–7.36)
Mining	15	127,560	0	–
Manufacturing	15	7,125,019	206	0.32 (0.23–0.45)
Other industry	15	469,731	42	1.00
Diffuse pleural thickening				
Construction	15	438,118	396	7.58 (5.73–10.03)
Mining	15	127,560	0	–
Manufacturing	15	7,125,019	221	0.33 (0.19–0.35)
Other industry	15	469,731	56	1.00

Note: *CI*: confidence interval; IRR: incidence rate ratio.

**Table 2.** Risk of asbestos-related diseases in the manufacturing industry ^A^.

	Observation period (years)*	Person-years	No. events	IRR (95% *CI*)
Lung cancer due to asbestos				
Chemical	16	553,151	229	0.40 (0.34–0.47)
Ceramic	16	619,994	373	0.58 (0.51–0.67)
Steel	16	767,556	167	0.21 (0.18–0.25)
Non-ferrous metal	16	284,123	36	0.12 (0.09–0.17)
Metal products	16	1,538,975	139	0.09 (0.07–0.11)
General machinery	16	1,025,843	180	0.17 (0.14–0.20)
Electrical machinery	16	439,597	54	0.12 (0.09–0.16)
Other transportation equipment	16	1,442,855	134	0.09 (0.07–0.11)
Shipbuilding and repairing	16	471,832	765	1.57 (1.41–1.76)
Other manufacturing	16	356,121	253	0.69 (0.59–0.80)
Other industry	16	489,094	504	1.00
Mesothelioma				
Chemical	16	553,151	233	0.24 (0.21–0.28)
Ceramic	16	619,994	347	0.32 (0.28–0.37)
Steel	16	767,556	229	0.17 (0.15–0.20)
Non-ferrous metal	16	284,123	18	0.04 (0.02–0.06)
Metal products	16	1,538,975	266	0.10 (0.09–0.11)
General machinery	16	1,025,843	372	0.21 (0.18–0.24)
Electrical machinery	16	439,597	133	0.17 (0.15–0.21)
Other transportation equipment	16	1,442,855	463	0.18 (0.17–0.21)
Shipbuilding and repairing	16	471,832	901	1.10 (1.00–1.21)
Other manufacturing	16	356,121	334	0.54 (0.48–0.61)
Other industry	16	489,094	849	1.00
Asbestosis				
Chemical	12	442,047	19	0.32 (0.19–0.53)
Ceramic	12	466,185	99	1.56 (1.12–2.17)
Steel	12	587,864	4	0.05 (0.02–0.14)
Non-ferrous metal	12	223,445	2	0.07 (0.02–0.27)
Metal products	12	1,236,400	18	0.11 (0.06–0.18)
General machinery	12	812,371	16	0.14 (0.08–0.25)
Electrical machinery	12	348,568	4	0.08 (0.03–0.23)
Other transportation equipment	12	1,152,204	26	0.17 (0.10–0.26)
Shipbuilding and repairing	12	362,160	56	1.14 (0.78–1.65)
Other manufacturing	12	289,312	39	0.99 (0.66–1.49)
Other industry	12	404,556	55	1.00
Benign asbestos pleural effusion				
Chemical	15	527,258	24	0.51 (0.31–0.84)
Ceramic	15	581,247	31	0.60 (0.38–0.95)
Steel	15	724,267	16	0.25 (0.14–0.44)
Nonferrous metal	15	270,723	2	0.08 (0.02–0.34)
Metal products	15	1,465,165	9	0.07 (0.03–0.14)
General machinery	15	974,264	14	0.16 (0.09–0.29)
Electrical machinery	15	418,032	3	0.08 (0.02–0.26)
Other transportation equipment	15	1,374,601	14	0.11 (0.06–0.21)
Shipbuilding and repairing	15	448,606	69	1.72 (1.17–2.52)
Other manufacturing	15	340,856	24	0.79 (0.48–1.30)
Other industry	15	469,731	42	1.00
Diffuse pleural thickening				
Chemical	15	527,258	0	0.00 (0.00–.)
Ceramic	15	581,247	52	0.75 (0.51–1.09)
Steel	15	724,267	14	0.16 (0.09–0.29)
Nonferrous metal	15	270,723	0	0.00 (0.00–.)
Metal products	15	1,465,165	8	0.05 (0.02–0.10)
General machinery	15	974,264	8	0.07 (0.03–0.14)
Electrical machinery	15	418,032	7	0.14 (0.06–0.31)
Other transportation equipment	15	1,374,601	14	0.09 (0.05–0.15)
Shipbuilding and repairing	15	448,606	78	1.46 (1.03–2.06)
Other manufacturing	15	340,856	17	0.42 (0.24–0.72)
Other industry	15	469,731	56	1.00

Note: ^A^ Different sectors in the manufacturing industry are based on the Japan Standard Industrial Classification (JSIC; Rev. 14, 2023) (see Supplementary Table S1). *CI*, confidence interval; IRR, incidence rate ratio.

## 4. Discussion

This study confirms that, in Japan, construction and shipyard workers have the highest risk of ARD. Our findings are derived from information for a relatively large number of the workers.

Similarly, the cohort studies by De Bono in Canada [24] and Järvholm in Sweden [25] have reported that construction workers have a higher incidence of ARDs that present in many occupational groups, whereas Walters' research [26] in the UK conducted a descriptive analysis assessing the proportion of asbestos exposure in construction associated with the onset of asbestosis cases. Plumbers, insulators, pipefitters, concrete workers, woodworkers, and electricians were the occupations with the highest risk within the construction sector in Canada and Sweden [24],[25], while in the UK, the occupation which identified as having the greatest risk were carpenters or joiners [26].

Other studies from Taiwan and Italy have reported links in shipyard workers between asbestos exposure and an increased risk of morbidity and mortality from ARDs [27],[28]. In Japan, several studies have found that the construction and shipyard industries are the highest ranked for developing ARD. For example, pleural plaques on chest computed tomography scans were primarily identified in construction workers diagnosed with lung cancer [29]. Other studies reported that lung cancer associated with plaque extent and pulmonary asbestos body content, as well as mesothelioma, were predominantly found among construction workers, followed by shipyard workers [30],[31]. In contrast, shipyard workers were found to be at the highest risk of developing diffuse pleural thickening (DPT), benign asbestos pleural effusion (BAPE), and autopsy cases of asbestosis followed by construction workers [32]–[34].

Another finding of our study was the increase in recent years of cases of occupational mesothelioma, with the greatest IR also observed among construction workers. A study by Gemba in Japan [31] and other various countries, such as Canada [24], Sweden [25], the US [35], and Italy [36],[37], have also reported that a high percentage of those with mesothelioma had worked in the construction industry, with asbestos the most frequently reported exposure. As a construction material, asbestos was extensively used before the 1970s [1],[20]. In Japan, spraying asbestos on ceilings, walls and iron frames in railroad coaches and structures became increasingly prevalent in 1957 as the use of asbestos increased in the construction industry [5],[6]. There was a notable increase in asbestos-containing building products between 1965 and 1975 [6], with building materials accounting for approximately 90% of asbestos use in Japan in 1995 [5],[7]. Occupational exposure to asbestos in the construction industry can still occur through building maintenance, repair, abatement, and demolition or through asbestos removal from buildings [1],[2],[4],[19],[20]. In Japan, it is possible that the risk of asbestos-related harm will persist into the future because the demolition of buildings containing asbestos is anticipated to peak around 2030 [7]. The rising incidence of mesothelioma in Japan is expected to peak somewhere between 2030 and 2039 [9].

In the shipyard industry, asbestos is commonly used for thermal insulation and surfacing materials in vessels [1],[27]. The main type of asbestos used in navy systems for insulation was amosite [6],[27]. In Japan, in the 1980s, asbestos was still present in 80% of ship materials because the majority of the ships that were being repaired had been built prior to 1975, when the use of sprayed asbestos was prohibited [6]. Therefore, lung cancer and mesothelioma among shipbuilders have been documented since the 1980s in Kure and Yokosuka, both of which have naval dockyards [6],[38]. The Japanese Government requested that the International Maritime Organization (IMO) revised the International Convention for the Safety of Life at Sea (SOLAS) to prohibit the use of asbestos-containing materials on all ships from 1 July 2002 [8].

In the ceramic industry, ARD compensation documents and the case reports in the study of Kishimoto et al. indicate that individuals involved in asbestos cement piping have developed mesothelioma [39]. Comparable experiences have been reported for workers involved in the manufacture of glass products (the use of asbestos gloves and asbestos ribbons), clay construction products (processing of wall materials containing asbestos and glass wool), and other ceramic and stone products (development and manufacture of asbestos products) [40]. According to the JSIC, all these industries are classified under the ceramic manufacturing group in Japan (major group 21) [21].

In the mining industry, the risk of ARDs is low compared with other industries. During World War II, raw asbestos could not be imported from overseas; the Japanese military searched for asbestos mines within Japan [5],[6], resulting in the operation of numerous tiny mines. However, the quality of the asbestos fibres mined was subpar, and the quantity produced was insufficient. Consequently, all asbestos mines in Japan closed before 1972 [6], explaining why the risk in this sector was low in the present study.

The Japanese government has attempted to regulate the risks associated with asbestos through several regulations. Beginning in 1975, the use of sprayed asbestos in buildings containing more than 5% asbestos by weight was prohibited [5],[6],[14]. Before then, the Pneumoconiosis Law of 1960 mandated that workers in dusty occupations undergo pneumoconiosis check-ups during employment [5],[14]. In 1995, the Industrial Safety and Health Law was revised to prohibit the use of crocidolite, amosite and materials containing more than 1% of either by weight [8],[14], and chrysotile-containing construction, friction, and adhesive materials were banned in 2004 [5],[8]. The Ordinance on Prevention of Asbestos Hazards (Ordinance of the Ministry of Health, Labour and Welfare No. 21) was enacted in 2005 to mitigate exposure to asbestos and other hazardous materials during demolition and building activities [5],[14]. In 2006, after the Kubota shock, the Japanese government banned items with more than 0.1% asbestos, and all asbestos products were banned in 2012. The Kubota shock also initiated the Act on Asbestos Health Damage Relief (Act No. 4 of 10 February 2006) to compensate people who were not covered by workers' compensation [5],[8],[14].

Today, almost all developed countries (~71 countries) have totally banned the use of asbestos [1],[4],[16]. Unfortunately, the extensive use of asbestos in developing countries, particularly in Asia, is expected to contribute to the emergence of new cases of ARD in the future [1],[7],[16],[17]. Consequently, countries that have not yet imposed a ban on asbestos should adopt a precautionary approach to its use, without waiting for an epidemic of ARDs to develop [7],[8].

This study has some limitations. First, the absence of sociodemographic data for each worker resulted in insufficient information regarding age, sex and other lifestyle data (e.g. smoking and alcohol consumption). Second, exposure measurement data from workplaces are lacking. Third, we cannot ascertain the precise time each patient was first exposed to asbestos in the workplace; thus, we are unable to discuss the latency period in this study. It is important to acknowledge that it is impossible to determine whether the documented ARDs were diagnosed in workers exposed before or after the implementation of various policies that reduced or banned asbestos use. Fourth, the cases of ARDs that have a lengthy latency period, such as mesothelioma, would only be able to be registered in the population at risk group only if former exposed workers continue to undertake health check-ups. Fifth, it is not clear from the data we used in this study whether the workers changed occupations during their exposure period. Finally, there is a possibility that the ARD cases might be underreported, particularly if certain employees may decline to undergo medical examinations. Nevertheless, we believe that the proportion of such cases is so small that the impact on this study is negligible. A strength of this study is that the estimates were derived using real-world data from large government databases. The IRRs we calculated are a useful tool for employers, clinicians, and decision makers to identify job categories at high risk of developing ARDs.

## 5. Conclusion

Despite the fact that the use of asbestos has been banned in Japan for the past 13 years, this study demonstrates that ARDs continue to occur in workers from a variety of industries, with those in construction and shipyards being most at risk. Therefore, workers who have previously been exposed to asbestos should be continuously monitored to identify any negative health effects. In addition, because asbestos is still present in our environment, professionals and the general public should be aware of diseases caused by asbestos.

## Use of AI tools declaration

The authors declare they have not used Artificial Intelligence (AI) tools in the creation of this article.


