# Supplementary material for: Significant risk of developing asbestos-related diseases in Japan's industries: An analysis of workers' compensation
Source: AIMS Public Health. 2025 Nov 4;12(4):1055–68. doi: 10.3934/publichealth.2025053 (PMC12795769; doi:10.3934/publichealth.2025053)
Supplement: Supplementary file 1 [file publichealth-12-04-053-s001.pdf]

Public Health

AIMS Public Health, 12(4): 1055–1068.
DOI: 10.3934/publichealth.2025053
Received: 29 July 2025
Revised: 19 September 2025
Accepted: 23 September 2025
Published: 04 November 2025

https://www.aimspress.com/journal/aimsph

Research article

Significant risk of developing asbestos-related diseases in Japan's industries: An analysis of workers' compensation

Leli Hesti Indriyati[1,2,*], Masamitsu Eitoku[1], Naw Awn J-P[1], Taro Tamura[1,3] and Narufumi Suganuma[1]

[1] Department of Environmental Medicine, Kochi Medical School, Kochi University, Kochi, Japan
[2] Department of Occupational Health, Faculty of Medicine, University of Muhammadiyah Prof. Dr. Hamka, Jakarta, Indonesia
[3] Izumino Hospital Medical Corporation Bouchikai, Kochi, Japan

* **Correspondence:** Email: lelihesti.indri@gmail.com; Tel: +0888802407.

Supplementary

Table S1. Manufacturing industry (Division E).

No	Major group	Group	JSIC code	ARD category
1.	Manufacture of food		E09	Other manufacturing
		Livestock products	091	Other manufacturing
		Seafood products	092	Other manufacturing
		Canned and preserved fruit and vegetable products	093	Other manufacturing
		Seasonings	094	Other manufacturing
		Sugar, starch, and saccharides	095	Other manufacturing
		Flour and grain mill products	096	Other manufacturing
		Bakery and confectionery products	097	Other manufacturing
		Animal and vegetable oils and fats	098	Other manufacturing
		Other foods and related products	099	Other manufacturing
2.	Manufacture of beverages, tobacco and feed		E10	Other manufacturing
		Soft drinks and carbonated water	101	Other manufacturing
		Alcoholic beverages	102	Other manufacturing
		Tea and coffee, except soft Drinks and carbonated water	103	Other manufacturing
		Manufactured ice	104	Other manufacturing
		Tobacco products	105	Other manufacturing
		Prepared animal foods and organic fertilizers	106	Other manufacturing
3.	Manufacture of textile products		E11	Other manufacturing
		Silk reeling, spinning, Chemical fibers and twisting and bulky yarns	111	Other manufacturing
		Woven fabrics	112	Other manufacturing
		Knit fabrics	113	Other manufacturing
		Dyed and finished textiles	114	Other manufacturing
		Rope, netting, lace and crude textile products	115	Other manufacturing

		Garments and shirts, except Japanese style	116	Other manufacturing
		Underwear	117	Other manufacturing
		Japanese style apparel, other textile apparel and accessories	118	Other manufacturing
		Other textile products	119	Other manufacturing
4.	Manufacture of lumber and wood products, except furniture		E12	Other manufacturing
		Sawing, planing and wood products	121	Other manufacturing
		Millwork, plywood and prefabricated structural wood products	122	Other manufacturing
		Wooden, bamboo and rattan containers	123	Other manufacturing
		Other manufacture of wood products, including bamboo and rattan	129	Other manufacturing
5.	Manufacture of furniture and fixtures		E13	Other manufacturing
		Furniture	131	Other manufacturing
		Furniture for religious Purposes	132	Other manufacturing
		Fixtures	133	Other manufacturing
		Other furniture and fixtures	139	Other manufacturing
6.	Manufacture of pulp, paper and paper products		E14	Other manufacturing
		Establishments engaged in administrative or ancillary economic activities (14 manufacture of pulp, paper and paper products)	140	Other manufacturing
		Pulp	141	Other manufacturing
		Paper	142	Other manufacturing
		Coated and glazed paper	143	Other manufacturing
		Coated and glazed paper	144	Other manufacturing
		Paper containers	145	Other manufacturing
		Other pulp, paper and paper products	149	Other manufacturing
7.	Printing and allied industries		E15	Other manufacturing

		Printing	151	Other manufacturing
		Plate making for printing	152	Other manufacturing
		Bookbinding and finishing	153	Other manufacturing
		Services related to printing	159	Other manufacturing
8.	Manufacture of chemical and allied products		E16	Chemical
		Chemical fertilizers	161	Chemical
		Industrial inorganic chemical products	162	Chemical
		Industrial organic chemical products	163	Chemical
		Oil and fat products, soaps, synthetic detergents, surface-active agents and paints	164	Chemical
		Medicines	165	Chemical
		Toiletries, toothpaste and toilet preparations	166	Chemical
		Other chemicala and allied products	169	Chemical
9.	Manufacture of petroleum and coal products		E17	Chemical
		Petroleum refining	171	Chemical
		Lubricating oils and greases (not made in petroleum refineries)	172	Chemical
		Coke	173	Chemical
		Paving materials	174	Chemical
		Other petroleum and coal products	179	Chemical
10.	Manufacture of plastic products, except otherwise classified		E18	Other manufacturing
		Plastic plates, bars and rods, pipes and tubes, pipe fittings and profile extrusions	181	Other manufacturing
		Plastic films, sheets, floor coverings and synthetic Leather	182	Other manufacturing
		Industrial plastic products	183	Other manufacturing

		Foamed and reinforced plastic Products	184	Other manufacturing
		Compounding plastic materials, including reclaimed plastics	185	Other manufacturing
		Other plastic products	189	
11.	Manufacture of rubber products		E19	Chemical
		Tires and inner tubes	191	Chemical
		Rubber and plastic footwear and its findings	192	Chemical
		Rubber belts and hoses, mechanical and industrial rubber products	193	Chemical
		Other rubber products	199	Chemical
12.	Manufacture of leather tanning, leather products and fur skins		E20	Chemical
		Leather tanning and finishing	201	Chemical
		Mechanical and industrial leather products, except gloves and mittens	202	Chemical
		Cut stock and findings for leather footwear	203	Chemical
		Leather footwear	204	Chemical
		Leather gloves and mittens	205	Chemical
		Baggage	206	Chemical
		Handbags and small cases	207	Chemical
		Fur skins	208	Chemical
		Other tanning leather Products	209	Chemical
13.	Manufacture of ceramic, stone and clay products		E21	Ceramic
		Glass and its products	211	Ceramic
		Cement and its products	212	Ceramic
		Clay products for construction, except those of pottery	213	Ceramic
		Pottery and related products	214	Ceramic

		Clay refractories	215	Ceramic
		Carbon and graphite products	216	Ceramic
		Abrasive and its products	217	Ceramic
		Aggregate and stone products	218	Ceramic
		Other ceramic, stone and clay products	219	Ceramic
14.	Manufacture of iron and steel		E22	Steel
		Iron industries	221	Steel
		Steel, with rolling facilities	222	Steel
		Steel materials, except made by smelting furnaces and steel works with rolling facilities, except coated steel	223	Steel
		Coated steel	224	Steel
		Ferrous metal machine parts and tooling products	225	Steel
		Other iron and steel	229	Steel
15.	Manufacture of non-ferrous metals and products		E23	Non-ferrous metal
		Primary smelting and refining of non-ferrous metals	231	Non-ferrous metal
		Secondary smelting and refining of non-ferrous metals, including non-ferrous alloys	232	Non-ferrous metal
		Rolling of non-ferrous metals and alloys, including drawing and extruding	233	Non-ferrous metal
		Electric wire and cable	234	Electrical machinery
		Non-ferrous metal machine parts and tooling products	235	Non-ferrous metal
		Other non-ferrous metal products	239	Non-ferrous metal
16.	Manufacture of fabricated metal products		E24	Metal products
		Tin cans and other plated sheet products	241	Metal products
		Tableware (occidental type), cutlery, hand tools and hardware	242	Metal products
		Heating and cooking apparatus, and plumbing supplies	243	Metal products

		Fabricated constructional and architectural metal products, including fabricated plate work and sheet metal work	244	Metal products
		Metal machine parts and tooling products	245	Metal products
		Metal coating, engraving and heat treating, except enameled ironware	246	Metal products
		Fabricated wire products, Except screws	247	Metal products
		Bolts, nuts, rivets, machine screws and wood screws	248	Metal products
		Other fabricated metal products	249	Metal products
17.	Manufacture of general-purpose machinery		E25	General machinery
		Boilers, engines and turbines	251	General machinery
		Pumps and compressors	252	General machinery
		General industry machinery and equipment	253	General machinery
		Other general-purpose machinery and machine parts	259	General machinery
18.	Manufacture of production machinery		E26	General machinery
		Agricultural machinery and equipment, except agricultural tools	261	General machinery
		Machinery and equipment for construction and mining	262	General machinery
		Textile machinery	263	General machinery
		Daily lives industry machinery	264	General machinery
		Basic material industry machinery	265	General machinery
		Metalworking machinery and its equipment	266	General machinery
		Semiconductor and flat-panel display manufacturing equipment	267	General machinery
		Other production machinery and machine parts	269	General machinery
19.	Manufacture of business oriented machinery		E27	General machinery
		Office machines	271	General machinery
		Service industry and amusement machines	272	General machinery

		Measuring instruments, analytical instruments, testing machines, surveying instruments and physical and chemical instruments	273	General machinery
		Medical instruments and apparatus, and medical supplies	274	General machinery
		Optical instruments and apparatus, and lenses	275	General machinery
		Ordnance and accessories	276	General machinery
20.	Electronic parts, devices and electronic circuits		E28	Electrical machinery
		Electronic devices	281	Electrical machinery
		Electronic part	282	Electrical machinery
		Storage media	283	Electrical machinery
		Electronic circuit	284	Electrical machinery
		Unit part	285	Electrical machinery
		Other electronic parts, devices and electronic circuits	289	Electrical machinery
21	Manufacture of electrical machinery, equipment and supplies		E29	Electrical machinery
		Electrical generating, transmission, and distribution apparatus	291	Electrical machinery
		Industrial electrical apparatus	292	Electrical machinery
		Household electric appliances	293	General machinery
		Electric bulbs and lighting fixtures	294	Electrical machinery
		Primary batteries (dry and wet)	295	Electrical machinery
		Electronic equipment	296	Electrical machinery
		Electric measuring instruments	297	Electrical machinery
		Other electrical machinery equipment and supplies	299	Electrical machinery
22.	Manufacture of information and communication electronics equipment		E30	Electrical machinery
		Communication equipment and related products	301	Electrical machinery

		Image and audio equipment	302	Electrical machinery
		Electronic data processing machines, digital and hybrid computer, and peripheral equipment	303	Electrical machinery
23.	Manufacture of transportation equipment		E31	Other transportation equipment
		Motor vehicles, parts and accessories	311	Other transportation equipment
		Railroad vehicles and parts	312	Other transportation equipment
		Shipbuilding and repairing, and marine engines	313	Shipbuilding and repairing
		Aircraft and parts	314	Other transportation equipment
		Industrial trucks and parts and accessories	315	Other transportation equipment
		Other transportation equipment	319	Other transportation equipment
24.	Other manufacturing industries		E32	Other manufacturing
		Precious metal products, including jewels	321	Other manufacturing
		Costume jewelry, costume accessories, buttons and related products, except precious metals and jewelry	322	Other manufacturing
		Watches, clocks, clockwork-operated devices and parts	323	General machinery
		Musical instruments	324	General machinery
		Toys and sporting goods	325	Other manufacturing
		Pens, lead pencils, painting materials and stationery	326	Other manufacturing
		Lacquerware	327	Other manufacturing
		Sundry goods of "tatami" mats, etc.	328	Other manufacturing
		Manufacturing industries, N.E.C.	329	Other manufacturing

AIMS Press © 2025 the Author(s), licensee AIMS Press. This is an open access article distributed under the terms of the Creative Commons Attribution License (https://creativecommons.org/licenses/by/4.0)
